# Correlation of Clinical, Endoscopic, and Pathological Findings among Suspected Peptic Ulcer Disease Patients in Abuja, Nigeria

**DOI:** 10.1155/2021/9646932

**Published:** 2021-06-30

**Authors:** Onyedika Godfrey Okoye, Oluwole Olayemi Olaomi, Alexander M. E. Nwofor, Paul Jibrin, Cephas Shallangwa Batta, Abubakar Gagarawa Yaú, Olawale A. Badejo

**Affiliations:** ^1^Department of Surgery, National Hospital Abuja, Nigeria; ^2^Department of Surgery, Nnamdi Azikiwe University Teaching Hospital, Nnewi, Nigeria; ^3^Department of Histopathology, National Hospital Abuja, Nigeria

## Abstract

**Background:**

Peptic ulcer disease (PUD) remains one of the most prevalent gastrointestinal diseases and has been linked to *Helicobacter pylori* (*H. pylori*) infection. This condition may be suspected on clinical grounds, but diagnosis is established using upper gastrointestinal endoscopy.

**Aims:**

To determine the correlation between the endoscopic and pathological findings among suspected PUD patients who have been referred for diagnostic upper gastrointestinal endoscopy in National Hospital Abuja.

**Methods:**

This is a hospital-based prospective study conducted among suspected PUD patients at National Hospital Abuja over a one-year period. Clinical, endoscopic, and histological findings were ascertained and documented. Data obtained were analyzed using SPSS version 21.0. Tests of significance were done using the chi-square test and Student *t*-test at 95% confidence intervals.

**Results:**

One hundred and thirty-two patients were included in the study. The ages ranged from 15 to 87 years, mean age 43.30 ± 11.94 years. Seventy-seven (58.3%) patients had abnormal endoscopic findings, of whom 37 (28.0%) had PUD. Prevalence of *H. pylori* infection was 42.2% and was found in 81.1% of PUD patients. *H. pylori* was significantly associated with confirmed PUD (*p* < 0.001) and abnormal endoscopic findings (*p* < 0.001). No association was found between normal endoscopic findings and histological findings (*p* = 0.924).

**Conclusion:**

There is a poor correlation between clinical and endoscopic diagnoses of PUD. *H. pylori* was found to be significantly associated with PUD and abnormal endoscopic findings. Endoscopic facilities should therefore be made available and accessible for proper PUD diagnosis. Empirical treatment of *H. pylori* in patients with diagnosed PUD is strongly recommended.

## 1. Introduction

Peptic ulcer disease (PUD) remains one of the most prevalent and costly gastrointestinal diseases [1]. Chronic and recurrent dyspeptic symptoms such as epigastric pain, postprandial fullness, and early satiety are common in the general population [2, 3]. In a systematic review of 31 published studies, the pooled incidence of uncomplicated PUD was approximately one case per 1000 person-years in the general population, and the incidence of ulcer complications was approximately 0.7 cases per 1000 person-years [4].

The discovery of *Helicobacter pylori* (*H. pylori*) in the human stomach and its association with PUD in 1983 has thrown more light on the pathogenesis of the disease and influenced its management [5, 6]. *H. pylori* chronically infects more than half of the world's population, with a prevalence rate ranging from 25% in developed countries to over 90% in developing countries [7]. It is recognized to be associated with several upper gastrointestinal pathologies such as chronic gastritis, peptic ulceration, mucosal associated lymphoid tissue (MALT) lymphoma, and gastric carcinoma [7, 8, 9]. *H. pylori* might be involved in the pathogenesis of many extra gastrointestinal tract diseases [10]. Gastric ulcers are known to be a marker of an increased risk of gastric cancer, primarily because both diseases are related to underlying gastritis [11, 12]. It has been shown that populations with 100% *H. pylori* infection rate have about 6-fold increased risk of gastric cancer compared with those that have no infection. It has also been estimated that the risk of gastric cancer attributable to *H*. *pylori* is 80% in developing countries [13]. A major complication of PUD in Africa is gastric outlet obstruction; most cases are usually due to the chronic duodenal ulcer with scarring [14].

Although *H. pylori* infection and nonsteroidal anti-inflammatory drugs have for long been considered to be the major aetiological factors in the causation of PUD [15], a review of medical literature suggests that the proportion of *H. pylori*-negative PUD has been increasing in developed countries [15]. PUD cannot be diagnosed accurately based on symptoms alone, as endoscopic investigation in the form of oesophago-gastro-duodenoscopy (OGD) is required to establish a diagnosis [16]. The recommended primary therapy for *H. pylori* infection is proton pump inhibitor-based triple therapy [17]. These regimens result in a cure of infection and ulcer healing in approximately 85–90% of cases [18].

Several works have been done on PUD and *H*. *pylori* in different regions of Nigeria including South West [19], North East [20], and North West [21]; a careful search of the literature did not show much work from North Central Nigeria.

It is also important to identify the peculiarity of the study group in this work. Whereas most works from Nigeria were done on dyspeptic patients who are heterogenous and nonspecific, this work is limited to patients suspected to have PUD on clinical grounds.

Therefore, this study will serve as a background work in this group of patients in this part of the country and will be used to compare other works in other regions.

## 2. Methods

This is a prospective clinical and laboratory-based study to determine the clinical, endoscopic, and pathological correlation among suspected PUD patients referred for diagnostic OGD at National Hospital Abuja, Nigeria.

National Hospital Abuja is a tertiary healthcare delivery centre located in North Central Nigeria. It is a referral centre and receives patients from Abuja and beyond.

This study was done over a 12-month period from June 2016 to June 2017. The participants include consecutive patients referred by the gastroenterology unit of our internal medicine department, patients from other surgical units, patients referred by family physicians, and those referred from private and general hospital around Abuja and environs where endoscopy facilities are not available. The patients studied were stable patients who came to the hospital as outpatients. All adult patients referred for diagnostic upper gastrointestinal endoscopy on suspicion of PUD based on clinical features were included. However, patients who presented with complications of PUD including bleeding, perforation, and gastric outlet obstruction; patients with prominent reflux symptoms; and patients who did not consent for the study were excluded. Sample size was calculated at 95% confidence level using the prevalence of PUD in Nigeria as 38.7% [19].

Permission for this study was obtained from the National Hospital Abuja research and ethics committee. Patients who fulfilled the inclusion criteria were asked to give informed consent in writing.

Selected patients were made to undergo OGD after an overnight fast. Oropharyngeal 10% xylocaine spray was given for local anaesthesia. Patients were placed in left lateral position with a mouth guard to protect the endoscope. Endoscopic examination was undertaken under patient monitor using a fibre optic Karl Storz endoscope (model 20090331) according to standard protocol.

Under direct vision, the endoscope was passed through the pharynx, the oesophagus, and the stomach and into the duodenum, with careful inspection during both insertion and slow withdrawal. Air was insufflated to distend the lumen and aid in movement and viewing. Liquid was aspirated through the suction channel. The fundus was inspected using a “J”–manoeuvre (retroversion).

During endoscopy, three gastric antral mucosal biopsies were obtained for histology and *H. pylori* testing using a histopathological method where haematoxylin/eosin and Giemsa stain were used. Abnormal and suspicious lesions were also biopsied. The procedures were carried out by four consultant general surgeons including the principal investigator. The histological analysis was done by a consultant histopathologist. The symptomatology and demographic characteristics of the patients were obtained by the researcher using interviewer-administered questionnaires.

Data recording and processing were done using SPSS (Statistical Package for Social Sciences) version 21.0. A value of *p* < 0.05 was considered significant. The chi-square test (*x*^2^) and Student *t*-test were used to test the differences between the qualitative and quantitative variables. Frequency tables and charts were used where necessary. A few patients did not submit their samples and were excluded from the study.

## 3. Results

**Figure 1:**
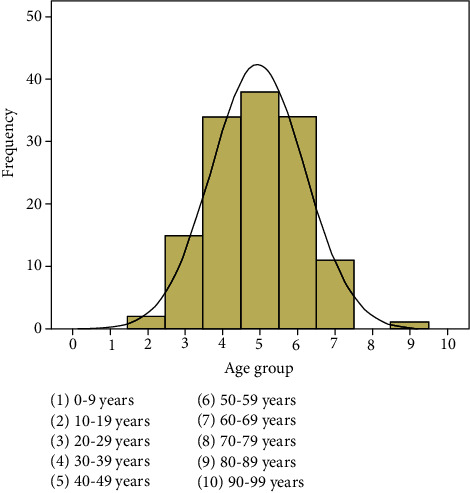
Age group distribution of the study population.

A total of 139 patients were initially recruited into this study over a period of 12 months (June 2016 to June 2017), but only 132 patients were eligible for analysis, giving an attrition rate of 5.04%. The patients were composed of equal numbers of males and females (66 patients (50%) each). The mean age of the study group was 43.30 years (±11.94). The mean age for the males was 40.23 (±11.39) while that of females was 46.38 (±11.78). The females were significantly older than the males (*p* = 0.003). The age range of the study group was 15 to 87 years with the median age of 42 years. This is depicted in Figure 1 which revealed that the fifth decade of life constituted the largest age group making up 28.8% of the entire population.

Epigastric pain was the most common presenting symptom accounting for 94.7% of respondents. Other symptoms (postprandial fullness (1.5%) and early satiety (3.8%)) make up 5.3%. Similarly, no sign was demonstrable on abdominal examination in 94.7% of the patients while epigastric tenderness was found in 5.3% of cases.

**Figure 2:**
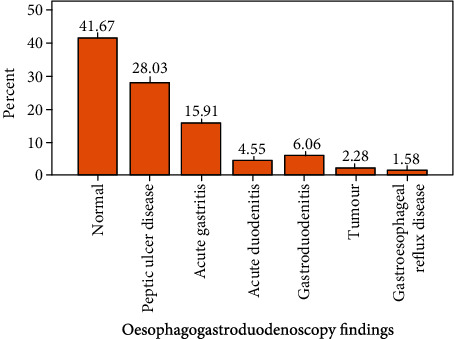
OGD findings of the study group.

As Figure 2 shows, normal study was the commonest finding on OGD constituting 41.7% of the findings, followed by PUD (28%), then acute gastritis (15.9%), gastroduodenitis (6.1%), acute duodenitis (4.5%), tumours (2.3%), and gastrooesophageal reflux disease (GERD) the least finding making up 1.5% of the entire findings. The three patients with gastric tumour were aged 42, 52, and 60 years. Out of 37 (28%) patients with OGD finding of PUD, duodenal ulcer patients were 25 (18.9%) and gastric ulcer were 12 (9.1%); thus, duodenal ulcer was about 2.1 times more frequent than gastric ulcer in this study.

The males diagnosed with PUD on OGD were 28 while the females were 9 giving a male to female ratio of 3 : 1, and this is statistically significant (*p* = 0.003). The mean age of PUD males was 42.0 ± 10.68 years while that of females was 40.62 ± 12.38 years. However, there is no significant difference in this age observation. The commonest age group was found to be in the fifth and the sixth decade.

Out of 25 patients with duodenal ulcer, 18 were males and 7 were females giving a male to female ratio of 3 : 1. However, of 12 patients with gastric ulcer, 5 were males and 7 were females giving a male to female ratio of 1 : 1.4.

**Figure 3:**
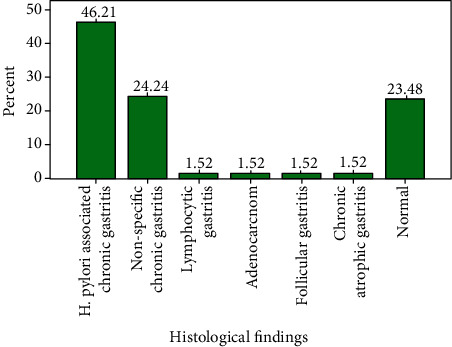
Histological findings of the study group.

Figure 3 shows that *H. pylori*-associated chronic gastritis was the commonest histological finding making up to 61 (46.2%), followed by nonspecific chronic gastritis 32 (24.2%) and normal histology 31 (23.5%). Other histological findings were adenocarcinoma, lymphocytic gastritis, follicular gastritis, and chronic atrophic gastritis each constituting 2 (1.5%) of the entire study group.

**Figure 4:**
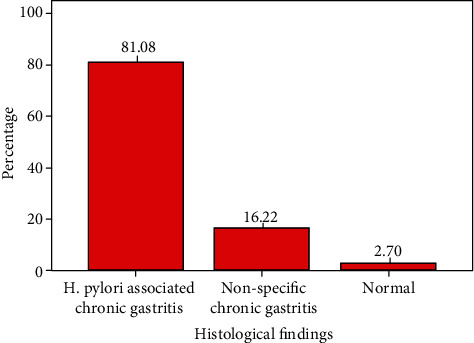
Histological findings in PUD patients.

Patients with OGD diagnosis of PUD were found to be positive for *H. pylori* in 81.1% of cases while nonspecific chronic gastritis and normal histology were found in 16.2% and 2.7% of cases, respectively (see Figure 4).

**Figure 5:**
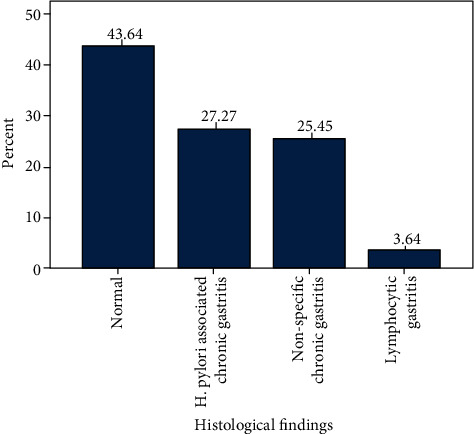
Histological findings in patients with normal study on OGD.

In patients with normal OGD findings, normal gastric mucosa was the commonest histological finding making up to 24 (43.6%) of the group followed by *H. pylori*-associated chronic gastritis 15 (27.3%), nonspecific chronic gastritis 14 (25.5%), and lymphocytic gastritis 2 (3.6%). This is shown in Figure 5. Though abnormal histological findings were more than the normal findings in this group of patients, the observed difference was not statistically significant (*p* = 0.924).

It is important to note that *H. pylori* positivity was significantly higher in PUD patients (81.1%) than in normal findings (27.3%) (*p* < 0.001, risk ratio = 2.9189, odds ratio = 11.1423). Similarly, patients with abnormal OGD findings have more frequency of *H. pylori*-associated chronic gastritis than those with normal OGD findings (*p* < 0.001). One hundred and two (77.3%) patients had received PUD treatment in the form of triple therapy in the past one month, while 30 (22.7%) had no previous treatment with triple therapy.

## 4. Discussion

Of the 139 patients initially recruited for the study, 7 could not complete the study protocol and were excluded from the analysis. This study analysis was based on 132 patients with clinical suspicion of PUD who were referred for upper gastrointestinal endoscopy.

The males and females recruited in this study were equal in number, 66 each. This may be viewed as a reflection of the general population of the federal capital territory where male population approximates that of the female. This 1 : 1 ratio was also found in a Lagos hospital-based study [22]. The absence of sex predilection among dyspeptic patients is buttressed by the findings of other workers in Ile Ife [23], Kano [20], and in England/Scotland [24]. The mean age 43.30 ± 11.94 years and the modal age group (fourth and fifth decade of life) in the studied group agree fairly with many other studies [22, 23, 25].

Females were significantly older than males in the studied group (*p* = 0.003). This may be related to the higher life expectancy of females in Nigeria and indeed in most parts of the world [26]. This infers that more elderly women are likely to present to the hospital than the elderly men generally. Another possible explanation is that postmenopausal women have lost the gastric protection of the estrogen/progesterone and hence are likely to present with dyspepsia at a later age.

The commonest endoscopic finding in this study was normal finding (41.7%). This is most likely due to possible functional dyspepsia in the studied population. Previous/ongoing treatment found in the majority of the patients may be contributory. Similar observations were documented in different parts of the world including the United States [27], Norway [28], Ghana [25], and Lagos Nigeria [22]. In contrast, Agbakwuru and his group in Ile ife [23], Jeje and his coworkers in Lagos [29], and a certain Canadian study [30] documented acute gastritis, duodenal ulcer, and reflux oesophagitis, respectively, as their commonest endoscopic findings though these studies were carried out on dyspeptic patients generally.

PUD (the second commonest endoscopic finding and the commonest abnormal finding) was found in only 28% of cases. This reveals a poor endoscopic agreement considering that all the patients were assumed to have PUD on clinical grounds. This shows that epigastric pain/discomfort is not specific to peptic ulcer disease alone. Oesophagitis may be a possible cause of epigastric pain [31]. Similarly, excluding reflux symptoms (as in this study) will exclude some PUD patients as demonstrated in previous studies [27, 28]. Another likely explanation is the high rate of previous treatment which may have resulted in ulcer healing. This fact has been substantiated in the literature where ulcer healing rate from 73 to 95% has been demonstrated with triple therapy [32].

This low finding of PUD on OGD is quite similar to findings elsewhere in Nigeria [22], Sweden [33], Canada [30], and Asian population [34]. Furthermore, this poor agreement between the clinical and endoscopic diagnoses was previously demonstrated by Agbakwuru and his co-workers in Ile Ife Nigeria [23] and Moayyedi and his colleagues in the United States [35].

The very low endoscopic finding of oesophageal reflux (1.5%) is not surprising given that patients with predominant reflux symptoms were excluded from this study. The fact that oesophageal reflux was identified despite the exclusion shows that it can present without typical reflux symptoms and may present with epigastric pain alone as earlier shown [31].

The gastric tumour found in the minority (2.3%) of the suspected PUD patients underscores the need for endoscopic confirmation of PUD to exclude malignancy especially in patients above the age of 40 years. This also shows that gastric malignancy may not present with alarm symptoms initially.

Duodenal ulcer was more frequent than the gastric ulcer in this study. This is in line with the global trend of PUD epidemiology. The male to female ratio of 3 : 1 is similar to findings in other centres [19, 36]. The males are significantly more affected than the females. This may be reflective of the relative protection conferred on premenopausal females by the estrogen/progesterone effect. However, there is no significant difference in the modal age group between the males and the females. This is because PUD is most prevalent in the fourth and fifth decades of life irrespective of the gender.

The prevalence of *H. pylori* in the studied group was 46.2%. This is relatively low and may be attributed partly to the high proportion of patients who had normal endoscopic findings. Other possible reasons include the small sample size, specimen handling, improved hygiene in Abuja, and more importantly the high proportion of patients with previous triple therapy (77.3%) in this study. The bacterial eradication has been shown to reduce the relapse rate from 85% to 0-20% in the United Kingdom [32].

This low prevalence rate of *H. pylori* in this work is similar to findings in Lagos (41%) [22], in Yola (56.7%) [37], in Ilorin (49.1%) [38], and in Ibadan (22.4%) [39]. However, the finding sharply differs from other centres where the prevalence in the studied population was high, up to 73 to 84% in Nigerian studies [19–21] and up to 75 to 85% in other African countries [40–42]. These differences in different regions affirm the relative importance of environmental factors in *H. pylori* epidemiology as has been demonstrated in the past [43].

The other histological findings depicted in this study closely mirror the findings in a related Nigerian study where nonspecific chronic gastritis was the commonest histology [22].

The prevalence of *H. pylori* among confirmed PUD patients in this study was high (81.1%). This is in line with the known strong association between *H. pylori* and PUD. This is strongly corroborated by the prevalence of 82 to 100% in South West Nigeria [19] and 90.9 to 95.8% in northern Nigeria [21].

The difference in *H. pylori* positivity between the PUD confirmed patients and normal findings on OGD seen in this study is statistically significant. Though this finding is expected, it differs from findings in North America where up to 50% of peptic ulcers were *H. pylori* negative [44]. Furthermore, higher association of *H. pylori* with abnormal OGD findings than the normal findings which is also statistically significant in this study portrays the association of *H. pylori* with other nonulcer gastroduodenal conditions including gastritis, duodenitis, and gastric malignancy. This has also been documented in a recent Indian study [45]. This difference in *H. pylori* association between abnormal and normal OGD findings has similarly been shown to be significant in a study by Hameed and his group [22] but surprisingly shown to be insignificant in another Lagos hospital-based study [29].

Normal gastric mucosa was the commonest histology (43.6%) in patients with normal OGD. These normal OGD patients also have *H. pylori* prevalence of 27.3%. This shows that *H. pylori* can be found in patients with no abnormal endoscopy findings. The reason for this is that *H. pylori* is said to be acquired early in life via faecooral means and therefore can exist in the stomach even in the absence of obvious pathology. This further affirms the fact that only 10 to 20% of individuals worldwide infected with *H. pylori* actually develop peptic ulcer [36].

Epigastric pain was the commonest presenting symptom in this work accounting for 94.7% of cases. This is probably due to the prior exclusion of patients with predominant reflux symptoms in this study. This finding is similar to the experience in Lagoon Hospital Lagos despite inclusion of the reflux symptoms [29].

Normal abdominal examination was the commonest sign on the study group. This may be partly explained by the exclusion of those presenting with complications of PUD. The minority of patients presenting with epigastric tenderness (5.3%) are likely to be those with acute gastritis with equally low proportion.

Majority of the patients (77.3%) has had previous triple therapy. This is because these patients had earlier seen a medical practitioner who probably started the medications before referral for endoscopy. The frequent patronage of a patent medicine store before hospital visit seen in this part of the world cannot be ruled out. The factors responsible for the high prevalence of *H. pylori* in PUD patients despite this treatment are subjects for future research. Perhaps, wrong prescription in terms of dosage and duration, poor monitoring, and compliance as well as substandard drugs, should be considered in the future work.

## 5. Conclusion

In conclusion, this study has shown the poor correlation between the clinical and endoscopic diagnosis of PUD (only 28% of the suspected PUD patients were confirmed on endoscopy). Normal study was the commonest endoscopic finding (41.7%) while peptic ulcer disease (28%) was the commonest abnormal finding. The observed prevalence of *H. pylori* was 46.2% and 81.1% in the entire studied group and among confirmed PUD patients, respectively. *H. pylori* positivity was significantly associated with PUD on the one hand and with abnormal endoscopic findings on the other hand. It has equally shown that epigastric pain is not restricted to PUD alone and so endoscopy is vital for the diagnosis and exclusion of malignancy. Availability and use of endoscopic facilities should be encouraged despite the cost. Proper and monitored eradication therapy for *H. pylori* should be equally sustained. Finally, a population-based study with larger sample size is needed to address some unanswered questions raised by this small piece.

## Data Availability

The original data is available and can be provided whenever it is required.
